# Incorporating Statistical Test and Machine Intelligence Into Strain Typing of *Staphylococcus haemolyticus* Based on Matrix-Assisted Laser Desorption Ionization-Time of Flight Mass Spectrometry

**DOI:** 10.3389/fmicb.2019.02120

**Published:** 2019-09-13

**Authors:** Chia-Ru Chung, Hsin-Yao Wang, Frank Lien, Yi-Ju Tseng, Chun-Hsien Chen, Tzong-Yi Lee, Tsui-Ping Liu, Jorng-Tzong Horng, Jang-Jih Lu

**Affiliations:** ^1^Department of Computer Science and Information Engineering, National Central University, Taoyuan City, Taiwan; ^2^Department of Laboratory Medicine, Chang Gung Memorial Hospital at Linkou, Taoyuan City, Taiwan; ^3^Ph.D. Program in Biomedical Engineering, Chang Gung University, Taoyuan City, Taiwan; ^4^Department of Information Management, Chang Gung University, Taoyuan City, Taiwan; ^5^Research Center for Emerging Viral Infections, Chang Gung University, Taoyuan City, Taiwan; ^6^School of Life and Health Sciences, The Chinese University of Hong Kong, Shenzhen, China; ^7^Warshel Institute for Computational Biology, The Chinese University of Hong Kong, Shenzhen, China; ^8^Department of Bioinformatics and Medical Engineering, Asia University, Taoyuan City, Taiwan; ^9^College of Medicine, Chang Gung University, Taoyuan City, Taiwan; ^10^Department of Medical Biotechnology and Laboratory Science, Chang Gung University, Taoyuan City, Taiwan

**Keywords:** *Staphylococcus haemolyticus*, strain typing, MALDI-TOF MS, Fisher's exact test, machine learning

## Abstract

*Staphylococcus haemolyticus* is one of the most significant coagulase-negative staphylococci, and it often causes severe infections. Rapid strain typing of pathogenic *S. haemolyticus* is indispensable in modern public health infectious disease control, facilitating the identification of the origin of infections to prevent further infectious outbreak. Rapid identification enables the effective control of pathogenic infections, which is tremendously beneficial to critically ill patients. However, the existing strain typing methods, such as multi-locus sequencing, are of relatively high cost and comparatively time-consuming. A practical method for the rapid strain typing of pathogens, suitable for routine use in clinics and hospitals, is still not available. Matrix-assisted laser desorption ionization-time of flight mass spectrometry combined with machine learning approaches is a promising method to carry out rapid strain typing. In this study, we developed a statistical test-based method to determine the reference spectrum when dealing with alignment of mass spectra datasets, and constructed machine learning-based classifiers for categorizing different strains of *S. haemolyticus*. The area under the receiver operating characteristic curve and accuracy of multi-class predictions were 0.848 and 0.866, respectively. Additionally, we employed a variety of statistical tests and feature-selection strategies to identify the discriminative peaks that can substantially contribute to strain typing. This study not only incorporates statistical test-based methods to manage the alignment of mass spectra datasets but also provides a practical means to accomplish rapid strain typing of *S. haemolyticus*.

## Introduction

*Staphylococcus haemolyticus* is one of the most significant species among the coagulase-negative staphylococci (CoNS), whose main ecological niches are skin and the human and animal mucous membranes (Becker et al., 2014). They are often the causative agents of septicemia, peritonitis, otitis, and urinary tract infections. In particular, the multidrug resistance, the early acquisition of resistance to methicillin, and various glycopeptide antibiotics by this species has troubled patients for many years (Froggatt et al., 1989; Hiramatsu, 1998). Strain typing of pathogenic *S. haemolyticus* forms an important part of the response to modern public health infectious disease outbreaks (MacCannell, 2013). For example, an outbreak of *S. haemolyticus* had been reported to be the cause of burn wound infections after a serious explosion event in Taiwan during June 2015 (van Duin et al., 2016; Chang et al., 2018). Rapid typing of *S. haemolyticus* facilitates the identification of the origin of infection, and allows rapid infection control when patients are critically ill. Consequently, a cost effective and rapid identification strategy that targets strain typing issues is essential and needs to be incorporated in routine clinical microbiology laboratory practices.

Whole-cell matrix-assisted laser desorption ionization-time of flight mass spectrometry (MALDI-TOF MS) is widely used in clinical microbiology laboratories worldwide. This is because MALDI-TOF MS allows rapid, reliable, and cost-effective identification of bacterial species (Vrioni et al., 2018; Wang et al., 2018c). The MALDI-TOF mass spectrum contains extensive information regarding the matter that constitutes microorganisms. In addition to the identification of bacterial species, MALDI-TOF MS has the potential to allow strain typing and/or antibiotic resistance profiling with high accuracy when machine learning methods are also implemented (Croxatto et al., 2012; Mather et al., 2016). Compared to the other strain typing methods, such as pulse-field gel electrophoresis and multi-locus sequence typing (MLST), analysis by MALDI-TOF MS to determine strain type is advantageous owing to its lower cost and rapid turn-around-time (Wang et al., 2018b). Strain typing via MALDI-TOF MS is promising; however, the subtle differences in MALDI-TOF MS spectra of different strains has hindered the introduction of this type of analysis in a clinical context in the absence of incorporation of computational methods (Sandrin et al., 2013; Camoez et al., 2016). Numerous methods have been developed in recent years to overcome this drawback in strain typing by MALDI-TOF spectrum analysis. The visual examination of a MALDI-TOF pseudo-gel or spectrum to pinpoint strain-specific peaks has been implemented by some research groups (Wolters et al., 2011; Josten et al., 2013). Visual examination of the MALDI-TOF MS is easy in practice, but the analytical accuracy is highly dependent on the operator. Inter-batch and/or intra-batch analytical variation is extremely likely. Moreover, visual examination of a MALDI-TOF MS or pseudo-gel is labor-intensive. Analyzing complex proteomic data, such as those obtained by MALDI-TOF MS, by visual examination often does not attain the appropriate level of precision, adequate objectivity, and/or a high enough throughput.

With the rapid advancements in artificial intelligence, machine learning-based methods have been implemented to identify classifiers when facing such classification problems (Mather et al., 2016; Wang et al., 2018b). More specifically, the logistic regression (LR), support vector machine (SVM), the decision tree (DT), the random forest (RF), and k-nearest neighbor (KNN) approaches have been widely implemented to build classifier model systems. In recent years, the application of machine learning-based methods in the field of medicine has received considerable attention, and several studies have demonstrated that the use of artificial intelligence to analyze complex data in medical practice is apposite and promising (Shameer et al., 2018; Hannun et al., 2019). Specifically, machine learning-based classifiers allowing professional diagnosis of retinopathy (Gulshan et al., 2016), can be used to analyze electrocardiography data (Hannun et al., 2019), and have been used to predict the prognoses of diseases (Wang et al., 2016; Yu et al., 2016; Lin et al., 2018). In addition to image analysis, applying machine learning-based methods to proteomic studies, specifically MALDI-TOF MS investigations, has assisted in attaining high accuracy in strain type prediction and/or strain antibiotic resistance (Wang et al., 2018a,b,c). Machine learning-based methods are able to utilize the signal intensities of specific peaks in their predictions, and this provides additional and more improved information than those obtained by the traditional method based on the presence or absence of peaks (Walker et al., 2002; Wolters et al., 2011; Lasch et al., 2014). In addition to providing robust prediction accuracy, machine learning-based methods, when analyzing MALDI-TOF MS, are also able to generate sets of discriminative peaks that are essential for accurate prediction. These specific sets of discriminative peaks can be used to pinpoint the possible combinations of molecules that are responsible for the various strain types and the variation in drug resistance profiles (Vrioni et al., 2018).

As mentioned previously, slight differences in MALDI-TOF MS results among different strains should be considered critical in preprocessing the spectral data. Specifically, the determination or extraction of representative features is essential before constructing the classifiers. Yet, little research is being done to develop a definitive strategy to solve such issues, not to mention incorporating statistical tests. In this study, we first developed a statistical test-based strategy for dealing with the alignment issue for the MALDI-TOF MS according to the mass-to-charge ratio (m/z) values, and further considered the signal intensity to construct the classification models. Various machine learning algorithms were trained and validated with the aim of discriminating the ST3, ST42, and various other STs of *S. haemolyticus*. We also investigated the discriminative peaks that are central to strain typing of *S. haemolyticus* with MALDI-TOF MS. This approach will not only be beneficial in rapid outbreak control for *S. haemolyticus* infection but also provide a definite strategy for preprocessing the spectral data.

## Materials and Methods

### Bacterial Isolates

A total of 254 unique *S. haemolyticus* isolates had been collected at Chang Gung Memorial Hospital, Linkou branch, Taiwan. The period of collection was between June and November 2015, which was the period when a significant number of burn patients were admitted to the hospital. The isolates were stored at −70°C until use. This was a retrospective study investigating the relation between MS spectrum and microbial strain typing. No diagnosis or treatment was involved by the study. Waiver of informed consent was approved by the Institutional Review Board of Chang Gung Medical Foundation (No. 201600049B0).

### Analytical Measurement of MALDI-TOF MS

To carry out the analysis, we cultivated the isolates on blood agar plates (Becton Dickinson, MD, USA) initially in a batch manner. The isolates were cultured in 5% CO_2_ incubator for 16 h. We then conducted the analytical measurements required for MALDI-TOF MS following manufacturer's instructions. First, we picked a single colony from a blood agar plate and spread it onto a steel target plate as a thin film (Bruker Daltonik GmbH, Bremen, Germany). One μl of 70% formic acid (Bruker Daltonik GmbH, Bremen, Germany) was then applied onto the steel target plate followed by drying in room air. One μl of matrix solution (Bruker Daltonik GmbH, Bremen, Germany) was then added. After the sample preprocessing, a MicroFlex LT mass spectrometer (Bruker Daltonik GmbH, Bremen, Germany) using a linear positive model was used for data acquisition. For each batch, a Bruker Daltonics Bacterial test standard (Bruker Daltonik GmbH, Bremen, Germany) was analyzed to allow calibration. The sampling setting of the laser shot was 240 shots (20 Hz) for each isolate. The MALDI-TOF MS spectra were analyzed using Biotyper 3.1 software (Bruker Daltonik GmbH, Bremen, Germany). The analytical range of each spectrum was 2,000-20,000 m/z. *S. haemolyticus* identification was set at high confidence (score > 2 in the reports of Biotyper 3.1 software). Furthermore, FlexAnalysis 3.3 (Bruker Daltonik GmbH, Bremen, Germany) was also implemented to acquire the numerical spectra data which derived from MALDI-TOF MS. Specifically, the original signals were smoothed by Savitzky-Golay algorithm and their baselines were subtracted by the top hat method. Meanwhile, some thresholds that were adopted to extract reasonable peaks were setup as explained below: signal-to-noise ratio was 2, relative intensity and minimum intensity were both 0, maximal number of peaks was 200, peak width was 6, and height was 80%. On the basis of the single measurements, we hypothesized that strain typing of *S. haemolyticus* is possible when the variability issue is handled using information engineering technology.

### Multilocus Sequence Typing of *S. haemolyticus*

We defined the strain typing of *S. haemolyticus* by sequencing seven housekeeping genes, namely *arc, SH1200, hemH, leuB, SH1431, cfxE*, and *RiboseABC* (Panda et al., 2016). The sequencing results of these genes were used to assign the sequence types of *S. haemolyticus* throughout the present analysis using the MLST database (https://pubmlst.org/shaemolyticus/) powered by the BIGSdb genomics platform (Jolley et al., 2018).

### MS Data Preprocessing for Classifiers Construction

Several computational tools have been developed for the preprocessing and extraction of features from MS data (Wong et al., 2005; Mantini et al., 2007; Gibb and Strimmer, 2012). More specifically, spectral data preprocessing would transform a set of raw spectra into a numerical table which include mass-to-charge (m/z) states with associated intensity for each isolate. Generally, m/z values with adequate intensities are considered as the fingerprint signatures when using spectral data, and these can be extracted to build up models for discriminating different subgroups. Note that a peak has an m/z value. As a result, a valuable analysis would highly depend on the appropriate use of preprocessing techniques. The MS data derived from FlexAnalysis 3.3 were of high quality, but their resulting peaks were not aligned within the dataset. Meanwhile, the aforementioned tools lack of specific information about the reference spectrum when implementing the alignment of peaks. Therefore, we developed a statistical test-based method for determining the reference spectrum within a given dataset, then further realizing the alignment of the peaks.

The reference spectrum should be capable of discriminating between different subgroups within a dataset. Consequently, we mainly focused on determining what pattern of peaks in the reference spectrum can indicate the differences among different groups in this study. For each spectrum, we first rounded each m/z value to the nearest whole number, and then all peaks that occurred were used to form a set of named candidate peaks set (CPS). The peaks in CPS were then sorted into ascending order. After a tolerance value is suggested, each adjacent peak in the CPS is either lower than or is equal to the given tolerance value; in such circumstances, the one with the higher difference in occurring ratio is retained. The difference in occurring ratio for m/z = *k*, in Dalton (Da), is defined below.

$$\begin{array}{l} D_{k}=\frac{1}{3}\left\{\left|\frac{x_{1}}{n_{1}}-\frac{x_{2}}{n_{2}}|+|\frac{x_{1}}{n_{1}}-\frac{x_{3}}{n_{3}}|+|\frac{x_{2}}{n_{2}}-\frac{x_{3}}{n_{3}}\right|\right\}, \end{array}$$

where *x*_1_, *x*_2_, and *x*_3_ are the counts that are aligned to m/z = *k*, and *n*_1_, *n*_2_, and *n*_3_ are the number of isolates for ST3, ST42, and other ST types, respectively. For example, suppose that the tolerance value is 1 Da, and the CPS = (2428 Da, 2429 Da, 2435 Da, 2436 Da, 2437 Da, 2450 Da) with *D*_2428_ = 0.053, *D*_2429_ = 0.090, *D*_2435_ = 0.080, *D*_2436_ = 0.094, *D*_2437_ = 0.076, and *D*_2450_ = 0.120, then the final m/z values, 2429 Da, 2436 Da, and 2450 Da, are then used to create the representative peaks set (RPS), which has an ascending order. In other words, the RPS is the reference spectrum and feature set used to construct the classification models.

**Figure 1:**
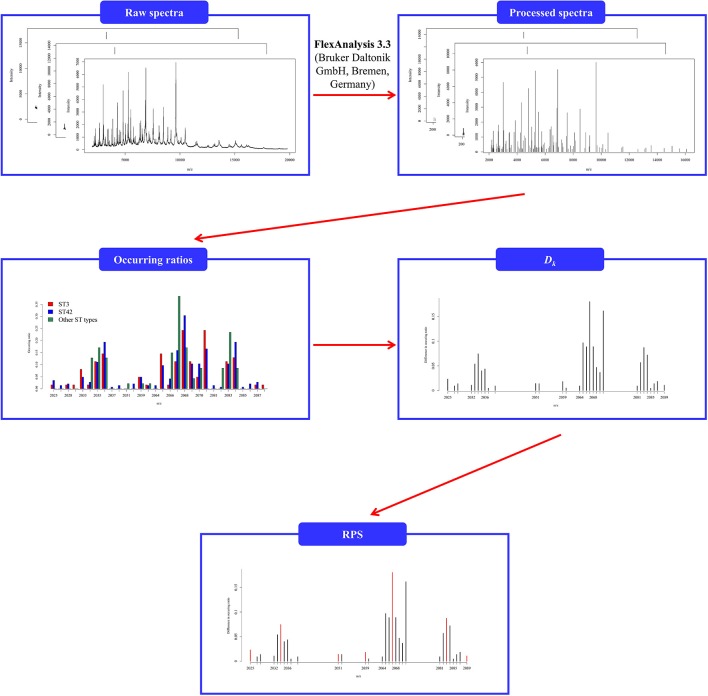
Flowchart of preprocessing of spectral data given that the tolerance value is 5. The incidence ratio was determined by the number of the isolates among the CPS. *D*_*k*_ was defined as the total difference between the incidence ratios.

To analyze the common peaks across the datasets given in this study, we employed Fisher's exact test (Raymond and Rousset, 1995) to determine a tolerance value for constructing the RPS due to relatively small sample sizes. For each tolerance value, there are three *p*-values determined by comparing ST3 and ST42, ST3 and other ST types, and ST42 and other ST types. As mentioned previously, the reference spectrum should be capable of discriminating between different subgroups within a dataset, and the tolerance value could be adopted according to its ability of separating these three groups. Therefore, the tolerance value was selected based on the obtained reference spectrum that would produce the largest number of *p*-values that were less than 0.001. We then further adopted the repeated 5-fold cross validation to demonstrate the efficiency of the determined tolerance value. Note that the determination of CPS and RPS was based on the training data when the repeated 5-fold cross validation was used. In other words, the repeated 5-fold cross validation was implemented here to simulate an external validation for evaluation of the performance in the determination of the reference spectrum. The flowchart of preprocessing is shown in Figure 1.

After determining the RPS, the alignment of the m/z with intensity is another critical part of the process, whereby the strength of signal at a specific m/z is determined. Therefore, in these circumstances, it is straightforward to move the specific m/z value of an isolate to the closest one in the RPS. As the tolerance value increases, more than one m/z values might be aligned to the same specific m/z in the RPS. In this situation, the intensity with the minimum distance between its own m/z and the specific m/z, is preserved. Hence, duplication problems can be solved. For instance, if both m/z = 2530 Da and m/z = 2535 Da in a spectrum are aligned to 2532 Da, which is a member of the RPS, the intensity of the m/z = 2530 Da is used for representing the strength of signal at 2532 Da. Supplementary Figure 1 illustrates how this alignment takes place.

### Development of Machine Learning-Based Classifiers

In this study, we implemented four machine learning methods; multiple logistic regression (MLR), support vector machine (SVM) learning, decision tree (DT) learning, and random forest (RF) learning, to construct the strain type classifiers for *S. haemolyticus* using R software (version 3.5.1, R Foundation for Statistical Computing, https://www.r-project.org/). MLR is a basic parametric model used in dealing with the present types of classification problems. The primary objective of SVM is to find a hyperplane that is able to segregate different classes of data and therefore it is commonly used to solve classification problems. DT and RF are both non-parametric tree-based strategies. Owing to the small size of data, the unsophisticated structure of DT can help us interpret the important features of the data more clearly. On the other hand, RF can provide evaluation metrics for the features and thus is able to identify the important features used during the model construction.

The glmnet package (Friedman et al., 2010) of R was applied during this study to construct the MLR model. More specifically, the MLR model can be defined as

$$\begin{array}{l} P\left(G=k|X=x\right)=\frac{\exp\left(\beta_{0k}+\beta_{k}^{T}x\right)}{\sum_{j=1}^{K}\exp\left(\beta_{0j}+\beta_{j}^{T}x\right)} \end{array}$$

where *K* is the number of levels of the response variable, and *G* = (1, 2, …, *K*) is the set of levels. Note that this parameterization is not estimable due to identical probabilities. However, regularization is able to deal with this. Hence the MLR model can be obtained by maximizing the penalized log-likelihood

$$\begin{array}{l} \underset{{\left\{\beta_{0j},\beta_{j}\right\}}_{1}^{K}\in {\mathbb{R}}^{K\left(p+1\right)}}{\max}\left\{\frac{1}{N}\overset{N}{\underset{i=1}{\sum }}\log p_{g_{i}}\left(x_{i}\right)-\lambda \overset{K}{\underset{j=1}{\sum }}P_{\alpha }\left(\beta_{j}\right)\right\} \end{array}$$

where *p*_*j*_(*x*_*i*_) = *P*(*G* = *j*|*x*_*i*_), and *g*_*i*_ ϵ (1, 2, …, *K*) is the *i*th response. Therefore, MLR-based classifiers are able to be constructed by adopting this package.

The SVM classifier was built using the e1071 package (Chang and Lin, 2011). In this package, the multi-class problem is approached via the “one-against-one” approach (Knerr et al., 1990). Consequently, there are *K*(*K*-1)/2 classifiers that are needed to be constructed for *K* classes. In this study, the SVM-based classifier was required to construct three classifiers due to the presence of three classes. More precisely, the training data was used to form the *i*th and *j*th classes and was able to deal with the following two-class classification problem.

$$\begin{array}{l} \underset{w^{ij},\;b^{ij},\;\xi^{ij}}{\max}\left\{\frac{1}{2}{\left(w^{ij}\right)}^{T}w^{ij}+C\underset{t}{\sum }{\left(\xi^{ij}\right)}_{t}\right\} \end{array}$$

subject to

$$\begin{array}{l} {\left(w^{ij}\right)}^{T}\phi \left(x_{t}\right)b^{ij}\geq 1-\xi^{ij},\;\text{if}\;x_{t}\;\text{in}\;\text{the}\;i\text{th}\;\text{class},\\ {\left(w^{ij}\right)}^{T}\phi \left(x_{t}\right)b^{ij}\leq -1+\xi^{ij},\;\text{if}\;x_{t}\;\text{in}\;\text{the}\;j\text{th}\;\text{class},\\ \xi^{ij}\geq 0. \end{array}$$

Following this, a voting strategy is adopted, the class with the maximum number of votes is considered to be the most probable one.

The DT-based classifier was implemented using the caret package (Therneau and Atkinson, 2018) of R. Specifically, the package mainly provides classification and regression trees (CART). Furthermore, the randomForest package (Liaw and Wiener, 2002) of R was also employed in this study to construct a random forest-based classifier. The package mainly provides an R interface using a Fortran program developed by Breiman (2001). Ensemble learning and bagging are the two important concepts used when creating the random forests. Furthermore, a random forest is a classifier consisting of a collection of tree-structured classifiers (Breiman, 2001). Therefore, according the voting results, we should be able to obtain the prediction for a specific data-set. In addition, RF provided the functions, that allow the evaluation of the effect of features during model construction. The mean decrease in accuracy and mean decrease in node impurity are provided by randomForest package (Liaw and Wiener, 2002). Note that the impurity is defined as

$$\begin{array}{l} I\left(p\right)=1-\overset{J}{\underset{i=1}{\sum }}p_{i}^{2} \end{array}$$

where *p*_*i*_ is the probability of correct classification.

In addition to the aforementioned multiclass classification approaches, we also adopted these methods when examining binary classification in order to better distinguish ST3 and ST42. The same package was implemented for this process, but in this case using the binary option. For instance, logistic regression (LR) was used to construct the binary classification model using the glmnet package (Friedman et al., 2010). Similarly, for SVM, DT, and RF, the same packages were adopted.

### Statistical Analysis

It is important to note that we were concerned not only with the frequency of the peaks, but also with the intensity of a specific peak among the multiple spectra, which is also a critical in discriminating these three groups. Therefore, in order to compare differences in intensities of specific peaks among these three groups, the Kruskal–Wallis test (Kruskal and Wallis, 1952) and Kendall's tau coefficient (Kendall, 1938) were both adopted as part of this study. Moreover, to obtain the ability of an individual peak to distinguish between the three groups, the area under the receiver operating characteristic curve (AUC) was taken into consideration. Note that to deal with multi-class performance evaluation, the pROC package (Robin et al., 2011) in R was implemented in order to obtain an estimation for the multi-class AUC (Hand and Till, 2001). When comparing the difference between two independent samples, the Wilcoxon rank-sum test was employed, and it was also implemented to compare cross validation performance. To find the optimal cut-off points for each ROC curve during binary classification, the OptimalCutpoints package (López-Ratón et al., 2014) was applied.

### Evaluation Metrics of the Classifiers

To evaluate the performance of the classifiers constructed by the aforementioned machine learning methods, the stratified 5-fold cross validation technique was implemented. The first procedure of the stratified 5-fold cross validation splits the dataset into 5 groups, preserving the percentage of data for each class. Then, one group is left as the testing dataset, while the remaining groups form the training dataset. The classification model was built according to the training dataset and was evaluated using the testing dataset. Note that each group was a testing dataset. Consequently, we obtained 5 prediction performances for these 5 groups. The average accuracy and the AUC among the five testing sets were determined in order to compare the performance when constructing the multiclass classifiers. As a result, the AUC was calculated by using the pROC package (Robin et al., 2011) in R. By way of contrast, we used sensitivity, specificity, accuracy, and AUC when evaluating the binary classification performance. More specifically, suppose that the class of ST42 is labeled as 1, these metrics are defined as follows:

$$\begin{array}{l} SN=\frac{TP}{TP+FN}\\ SP=\frac{TN}{FP+TN}\\ ACC=\frac{TP+TN}{TP+TN+FP+FN}, \end{array}$$

where TP means the true positives and refers to the number of ST42 that were correctly predicted by the classifier, TN means true negatives and refers to the number of ST3 that were correctly predicted by the classifier, FP means false positives and refers to the number of ST42 that were incorrectly predicted by the classifier, and FN means false negative and refers to the number of ST3 that were incorrectly predicted by the classifier.

### Feature Selection Strategies

In addition to applying the importance evaluation from RF, we also developed two strategies, the stepwise strategy and the forward strategy, to find the peaks that needed to be considered as classifiers. More specifically, these two strategies were adopted when constructing the multi-class RF-based classifiers in order to obtain the peaks that are essential when distinguishing these three groups.

The stepwise strategy starts initially with a specific peak, such as the one with the largest AUC, the largest absolute value of Kendall's tau coefficient, and so on. Further, the next peak to be selected must attain the largest AUC or accuracy when combined with the currently selected peak(s) among those peaks that have not been selected. The process is then repeated until the AUC or the accuracy does not increase anymore.

When using the forward strategy, the peaks must be sorted into a specific order. For example, the peaks can be sorted by their AUCs in the descending order. Then the forward strategy would follow this order to adding new peaks if the new one is able to increase the AUC or accuracy. Otherwise, the peak will not be regarded as a helpful feature when constructing the classifier, and thus will be discarded.

The sensitivity of both these strategies is dependent on the selection of the initial peak. In other words, the first selected peak will affect different peak combinations and this may produce different performances. Moreover, different criteria are likely also to result in different combinations. In this study, both AUC and the accuracy are two of the major concerns when building the multi-class classifiers. On the other hand, the balance between the sensitivity and specificity also needs to be taken into consideration. Nevertheless, the major aspects of the evaluation still are dependent on the AUC and the accuracy.

## Results

### Summary Statistics of Spectra Data

**Figure 2:**
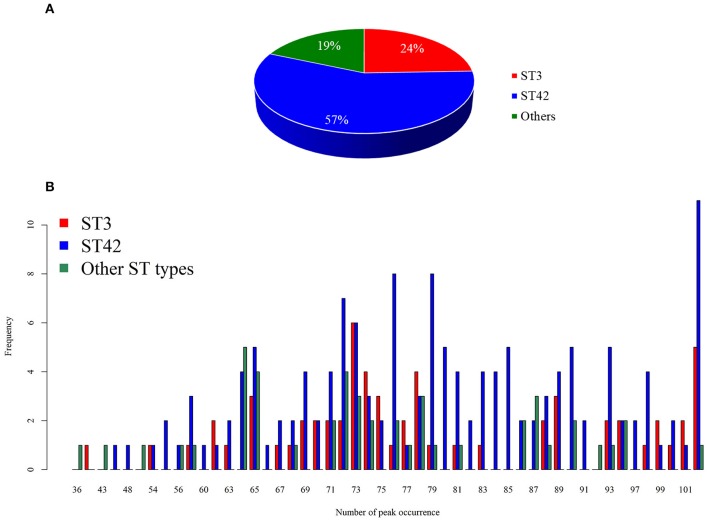
Distribution of the dataset. **(A)** Pie chart showing the distribution of dataset. **(B)** Number of identified peaks in each group.

Among the 254 isolates used in the present study, 62 isolates were ST3, 145 isolates were ST42, and 47 isolates were neither ST3 nor ST42 and formed a separate group of strains. The details of the other ST types show in Supplementary Table 1. Given that we aimed to develop and validate a rapid *S. haemolyticus* strain typing tool, we designed the classes based on the local epidemiology, whereas ST3 and ST42 accounted for the majority of strains. In clinical practice, the developed tool would provide preliminary strain typing information, notifying clinical physicians if the isolate of interest is of the major ST types. When the isolate of interest is classified by the model as a major ST type, outbreaks from the origin should be suspected and further investigation could be initiated immediately. As noted, this classification was determined by the local epidemiology of *S. haemolyticus* in Taiwan. Figure 2 demonstrates the data statistics and the distribution of number of peaks identified for each group. On an average, the number of peaks identified in the range 2,000 Da to 17,000 Da was 76.48, with a standard deviation of 13.46. More specifically, the average number of peaks identified for ST3 was 77.03, while that of ST42 was 77.68, and the number of peaks identified for the other ST types was 72.04. Although the number of peaks identified for the other ST types seemed to be lower than that for the other two strains, the Kruskal-Wallis rank sum test did not show a significant difference between the three groups (*p* = 0.0586). When spectra signal intensity was examined, the average (standard deviation) normalized intensity across the three groups was 0.16 (0.18). The average normalized intensity of ST3 was 0.13 (0.16), while that of ST42 was 0.17 (0.19), and that of the other group of ST types was 0.18 (0.18). The normalized intensity of ST3 seemed to be lower than that of other two groups and the result of the Kruskal-Wallis rank sum test also showed that there were significant differences between these three groups (*p* < 0.0001).

### Determination of Tolerance Value

**Figure 3:**
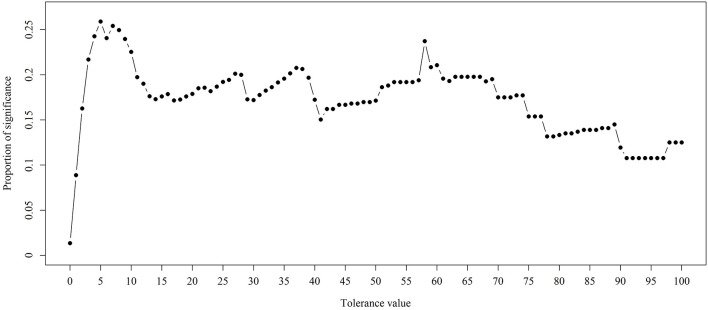
Proportion of significance for different tolerance values. Fisher's exact test was employed to examine the difference between two different ST types. The *p*-values were derived by the average of three *p*-values.

**Figure 4:**
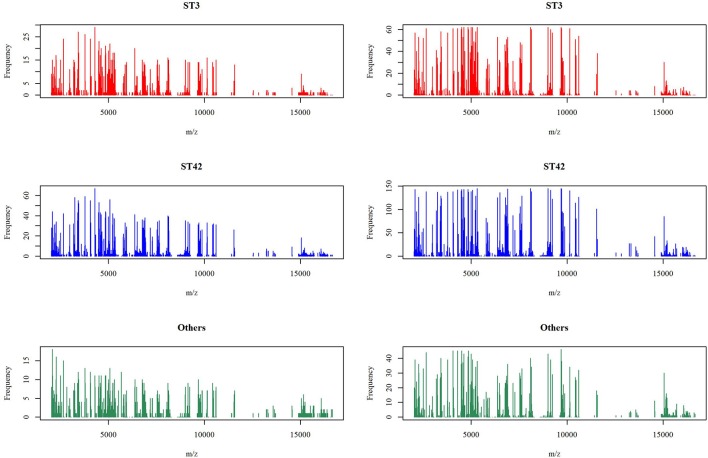
Mass spectra before and after peak alignment. The left panel is the number of spectra appearing the specific peaks under the original signal of the mass spectra and the right panel is after the alignment strategy with tolerance value 5.

**Figure 5:**
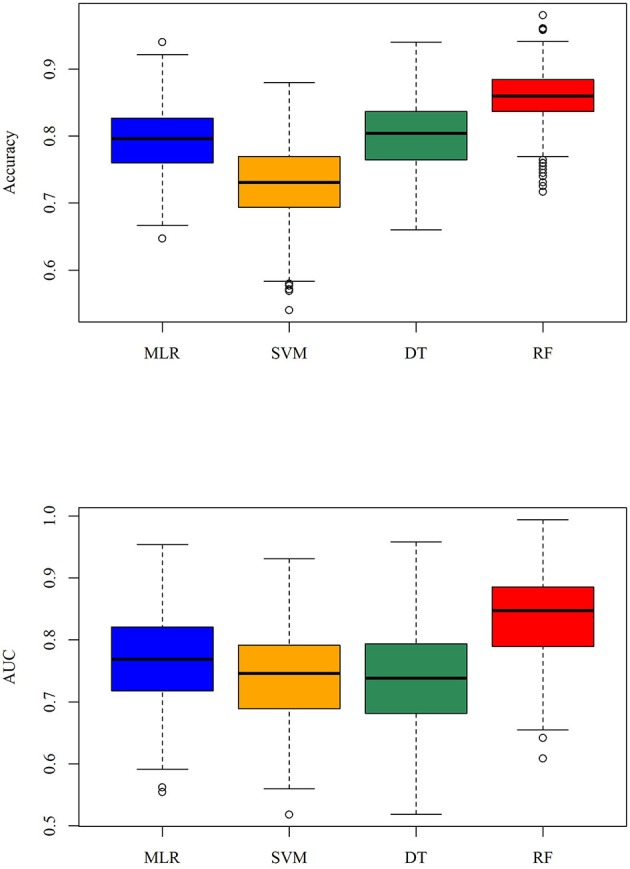
Boxplot of the accuracy and AUC for the repeated 5-fold cross validation when the tolerance value is 5.

**Figure 6:**
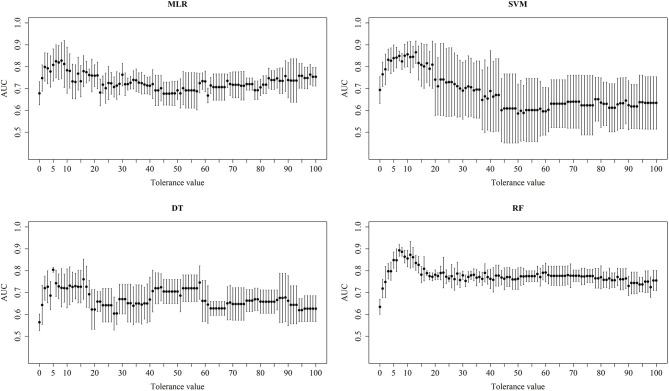
Performance of different classifiers. Mean and standard deviation AUC of the 5-fold cross validations for the different tolerance values using different machine learning methods.

In the previous section, we have described the strategy for determining the RPS using Fisher's exact test. Figure 3 demonstrates the proportion of significance for different tolerance values. More specifically, the proportion of significance was determined by the number of occurring significance. Note that the significance here indicates that the *p*-value of Fisher's exact test is <0.0001. When the tolerance value is 5, the proportion of significance is highest. The spectra with and without preprocessing is shown in Figure 4. In addition, Figure 5 demonstrates the performance of the 5-fold cross validation repeated 100 times. Specifically, there were 500 independent tests of ACCs and AUCs for evaluating whether the tolerance value was robust enough. These results implied that the tolerance value was adequate for further analysis. The AUC of different classifiers under different tolerance values, which are shown in Figure 6, demonstrated that the AUC was able to attain a value of 0.8 with a low standard deviation for the tolerance value of 5. Therefore, we used a tolerance value 5 for the feature selection because of its robustness. Table 1 shows the mean ± standard deviation of the accuracy and AUC values for the 5-fold cross validation using the different machine learning methods. Wilcoxon rank sum test was then used to compare their performances. It should be noted that the *p*-value next to the accuracy/AUC column is from the Wilcoxon rank sum test results and this was employed to compare the accuracy/AUC when using the MLR method on the test data during 5-fold cross validation. Furthermore, we also found that the RF values tended to be robust due to the presence of a lower standard deviation compared to other methods for the different tolerance values present in Figure 5. Hence the feature selection strategies, when implemented to find important features, used RF. It should be noted that the number of peaks in RPS was 583 for a tolerance value of 5 and thus it was these 583 features that were used to construct the multi-class classifiers used to discriminate the three groups.

**Table 1:** Performance of 5-fold cross validation.

	**Accuracy**	***p*-value**	**AUC**	***p*-value**
MLR	0.819 ± 0.028	–	0.808 ± 0.074	–
SVM	0.858 ± 0.029	0.0937	0.839 ± 0.060	0.5476
DT	0.840 ± 0.046	0.4005	0.804 ± 0.012	0.6905
RF	0.866 ± 0.014	0.0196	0.848 ± 0.037	0.3095

*Mean ± standard deviation accuracy and AUC of the 5-fold cross validations for the multiclass classifications using different machine learning methods when the tolerance value is 5. The p-values were derived by comparing with MLR. MLR, multiclass logistic regression; SVM, support vector machine; DT, decision tree; RF, random forest*.

### Results of Feature Selection Strategies on RF-Based Classifiers

Table 2 demonstrates the results of the two feature strategies when RF was used to construct the classification models. The forward strategy was highly dependent on the order of inclusion of the features. On the other hand, the starting peak in the stepwise strategy was critical. Both these strategies demonstrated that a reduction in the number of features appeared to increase the accuracy or AUC. In other words, the selected peaks were found to be highly correlated with *S. haemolyticus* and were able to distinguish between the three groups of ST strains.

**Table 2:** Performance of feature selection.

			**RF**		
**Strategy**	**Start up**	**# peaks**	**Accuracy**	**AUC**	***p*-value**
Stepwise	AUC	21	0.918 ± 0.024	0.921 ± 0.025	0.0079
	Kendall's tau	26	0.906 ± 0.008	0.897 ± 0.049	0.2222
	KW	20	0.902 ± 0.030	0.917 ± 0.042	0.0952
	IMP	27	0.910 ± 0.008	0.926 ± 0.020	0.0079
Forward	AUC	18	0.897 ± 0.032	0.896 ± 0.026	0.0952
	Kendall's tau	35	0.901 ± 0.024	0.893 ± 0.047	0.2222
	FE	25	0.902 ± 0.023	0.898 ± 0.035	0.0556
	KW	26	0.906 ± 0.031	0.902 ± 0.061	0.2222
	IMP-ACC	22	0.882 ± 0.032	0.864 ± 0.059	0.7533
	IMP-GINI	28	0.874 ± 0.034	0.836 ± 0.037	0.5476
No		583	0.866 ± 0.014	0.848 ± 0.037	-

*Mean ± standard deviation accuracy and AUC of the 5-fold cross validation of RF using different numbers of peaks selected by the forward and stepwise feature selection strategies using different orders of peaks and the corresponding performance using RF. AUC, area under the curve; FE, Fisher's exact test; KW, Kruskal-Wallis test; IMP-ACC, importance measure calculated by mean decreased accuracy using RF; IMP-GINI, importance measure calculated by mean decreased impurity using RF*.

**Figure 7:**
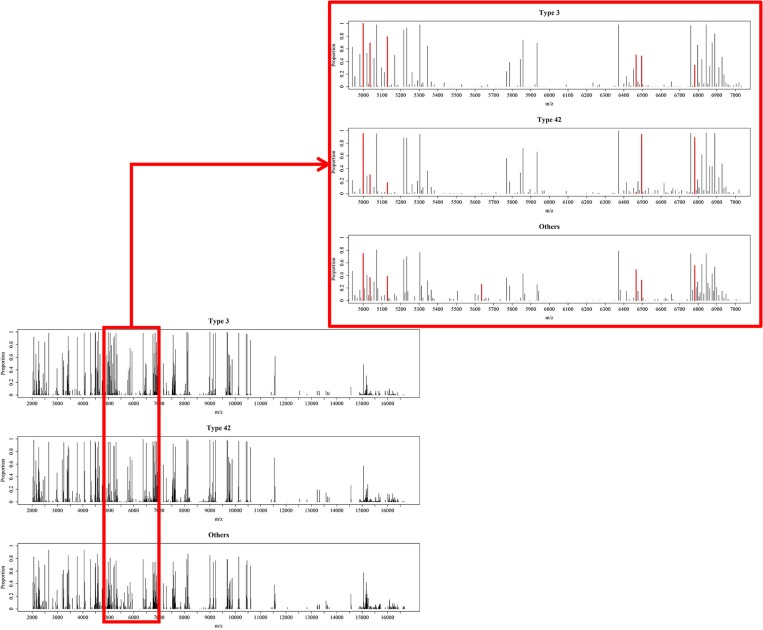
Overview of processed MS data. Occurrence proportions among the three groups over the range from 2,000 to 17,000 Da and zoomed in for the range 4,900 to 7,100 Da. The red areas include peaks 4548, 4673, 4999, 5036, 5129, 5635, 6466, 6496, and 6781, which are the important peaks when constructing the RF-based classifiers.

**Figure 8:**
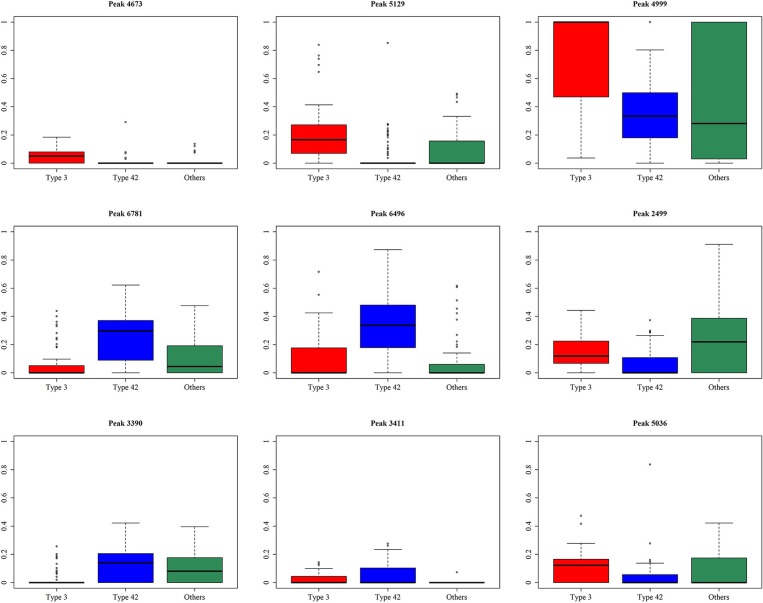
Boxplots for the normalized intensity for the discriminative peaks.

A total of 10 models were constructed by adopting different feature selection strategies and selecting different peaks. We next identified the peaks that were selected in more than five models and these were regarded as discriminative peaks. Table 3 shows the occurrence and proportions of these discriminative peaks. From this table it can be seen that the ST42 isolates almost always present the peaks 4999 and 6496, explicitly they were present in over 90% of samples. However, neither ST3 nor ST42 ever presented the peak 5635. In addition, Figure 7 presents the whole spectral incidence for the three groups, and specifically focuses on the area from 4700 to 7100 Da, which allows closer observation of the behavior of the discriminative peaks. Specifically, the red bars show the differences between these three groups that seem to be critical to constructing the classifiers. When considering the intensity, Table 4 presents the means and standard deviations of the normalized intensities of the discriminative peaks. Since the incidence tends to be small, and the normalized intensity is between 0 and 1, the average values also tend to be low. Nevertheless, some peaks still showed strong intensity. For example, peaks 6781, 6496, and 4999 still have relatively large intensity values. The Kruskal-Wallis test was employed to test difference among the three groups and when there was a difference between two groups, the *p*-value tended to be lower. Hence the *p*-values in Table 4 are very small. It should be noted that these discriminative peaks are the ones that are often selected using the various different feature selection strategies shown in Table 2. Moreover, the boxplots in Figure 8 can be used to demonstrate the distribution of intensities among the different ST types. According to Table 3, the intensity in event of a lower incidence tends to be smaller. This can also be seen in Figure 8 for peaks such as 4674 and 4659.

**Table 3:** Number of occurrence peaks (proportions) and average *p*-values using the Fisher's exact test for the discriminative peaks.

**Peak**	**Type 3**	**Type 42**	**Others**	***p*-value**
4673	40 (0.645)	5 (0.034)	5 (0.106)	0.022
5129	49 (0.790)	25 (0.172)	18 (0.383)	0.001*
4999	62 (1.000)	138 (0.952)	35 (0.745)	0.035
5635	0 (0.000)	0 (0.000)	12 (0.255)	0.333
6466	31 (0.500)	3 (0.021)	23 (0.489)	0.333
2499	52 (0.839)	59 (0.407)	33 (0.702)	0.035
3390	15 (0.242)	107 (0.738)	27 (0.574)	0.015
3411	20 (0.323)	70 (0.483)	1 (0.021)	0.015
5036	43 (0.694)	43 (0.297)	17 (0.362)	0.157
6496	30 (0.484)	136 (0.938)	15 (0.319)	0.039
6781	21 (0.339)	129 (0.890)	26 (0.553)	0.011

* *Indicated that the p < 0.01*.

**Table 4:** Means (standard deviation) and *p*-values using the Kruskal–Wallis test for the discriminative peaks.

**Peak**	**Type 3**	**Type 42**	**Others**	***p*-value**
4673	0.052 (0.049)	0.003 (0.026)	0.011 (0.032)	<0.001*
5129	0.209 (0.200)	0.031 (0.094)	0.094 (0.150)	<0.001*
4999	0.769 (0.307)	0.350 (0.236)	0.455 (0.427)	<0.001*
5635	0.000 (0.000)	0.000 (0.000)	0.126 (0.266)	<0.001*
6466	0.145 (0.185)	0.003 (0.025)	0.118 (0.155)	<0.001*
2499	0.151 (0.120)	0.057 (0.083)	0.222 (0.207)	<0.001*
3390	0.030 (0.062)	0.132 (0.110)	0.103 (0.111)	<0.001*
3411	0.024 (0.040)	0.054 (0.065)	0.002 (0.011)	<0.001*
5036	0.115 (0.102)	0.032 (0.082)	0.076 (0.109)	<0.001*
6496	0.108 (0.151)	0.338 (0.210)	0.089 (0.174)	<0.001*
6781	0.065 (0.119)	0.247 (0.162)	0.108 (0.126)	<0.001*

* *Indicated that the p < 0.01*.

### Classifier for Discriminating ST3 and ST42

Table 5 shows the performance of the classifiers used to distinguish ST3 and ST42. Since the majority of data available was for ST42, the specificity of these classifiers tended to be higher. Even so, the AUCs among the different classifiers also showed impressive results. In both Figures 7, 8, it can be seen that the incidence and intensities are evidently different for some specific peaks.

**Table 5:** Performance of binary classifier.

	**Sensitivity**	**Specificity**	**Accuracy**	**AUC**
LR	0.890 ± 0.062	0.968 ± 0.044	0.913 ± 0.045	0.919 ± 0.051
SVM	0.931 ± 0.055	0.983 ± 0.037	0.947 ± 0.032	0.969 ± 0.021
DT	0.938 ± 0.037	0.904 ± 0.032	0.928 ± 0.033	0.919 ± 0.024
RF	0.951 ± 0.031	1.000 ± 0.000	0.966 ± 0.022	0.972 ± 0.020

*Mean ± standard deviation sensitivity, specificity, accuracy, and AUC of 5-fold cross validation for binary class classification using different machine learning methods when tolerance value is 5. LR, logistic regression; SVM, support vector machine; DT, decision tree; RF, random forest*.

## Discussion

This is a study that focused on the strain typing of *S. haemolyticus* based on the MALDI-TOF MS utilizing statistical tests and machine learning methods simultaneously. Specifically, the Fisher's exact test was employed to determine the reasonable tolerance values on preprocessing the spectra data. We have not only constructed machine learning-based classifiers that allow for different feature selection strategies, but have also employed statistical tests to compare the performance of the various discriminative peaks related to the different ST types. The rapid identification of *S. haemolyticus* strain types will facilitate the identification of origins of infection and will also provide critically-ill patients with substantial benefits because it will allow for rapid infection control. Additionally, further exploration of the discriminative peaks will allow the identification of each corresponding peptide. Such findings should provide clinically valuable information pertaining to the different subtypes of *S. haemolyticus*.

Previous studies used “type templates” for each ST type based on the incidence of specific peaks in their MALDI-TOF MS spectra in order to handle the issue of peak shifting; furthermore, log-transformed intensity was used to represent corresponding signal strength for each peak (Wang et al., 2018a,b). These studies also used the signals with the highest incidence probability in a local region (± 5 m/z) as the center of each peak feature. In other words, determining the local region was based on the incidence probability without the adoption of any statistical tests. In this study, we used statistical analysis and also measured the performance of classifiers. Such an approach involving measurement of the tolerance value is an excellent approach for dealing with the peak shift problem present when using spectral data. As the tolerance value increases, the number of peaks in the RPS decreases, and vice versa. The reason is that a larger tolerance value may lead to the alignment of more discriminative peaks with the same specific peak. In contrast, a lower tolerance value results in a paucity of data. Specifically, in these circumstances, much less data can be aligned to the same specific peak, which produces a reduced amount of training data and eventually results in poor performance. In such circumstances we used both Fisher's exact test, and an evaluation of the variation in performance of different classifiers with different tolerance values. In short, the variation among different classifiers and tolerance values was taken into consideration and this increased the robustness of our model. When the tolerance value was 5, the significance value was the largest and the standard deviation among the 5-fold cross validation analysis tended to be lower. Therefore, we used 5 as the final tolerance value when creating the RPS using 583 peaks.

There are a variety of machine learning methods that can be used for modeling different types of data. In this study, we adopted a number of relatively uncomplicated models to construct the classifiers. These uncomplicated methods are readily interpreted, which makes interpretation of the peak results easier and allows the initiation of further investigations into specific peaks simpler. Multinomial logistic regression is a generalized logistic regression model that is used for handling multi-class problems and is one of the most common parametric statistical models. Our major concern in adopting the multinomial logistic regression model was multicollinearity. When the dependency among different independent variables is high, the estimators can be misinterpreted, and this may increase the prediction bias (Myers and Myers, 1990). Although the performance of MLR, as shown in Table 1, tended to be lower than other methods, the estimation of the parameters does seem to provide some information about the discriminative peaks. In other words, the estimators of the MLR were able to reveal which peaks potentially correlated with different ST types. It should be noted that a consideration of the standard errors of these estimators is an important reference point that can be used to avoid the multicollinearity effects. This is because there are few restrictions on the use of non-parametric methods such as SVM, DT, and RF. Their primary weakness is the time required for training the model when they use large scale datasets. However, this was not an issue in this study due to the relatively limited amount of data. Consequently, the performance of the non-parametric methods was better than that of MLR. Furthermore, the performance of RF was more robust than other methods. This is possibly due to two of the essential concepts of RF, namely ensemble learning and bagging. Previous studies also have reported the various advantages of RF (Boulesteix et al., 2012). In this study, we have also demonstrated that RF not only provides the highest accuracy and AUC, but it also retains the lower standard deviation.

Only a slight variation at the bacterial subspecies level is observed when they are compared using mass spectra (Lasch et al., 2014; Wang et al., 2018b). Nevertheless, until now, no studies have been able to identify the discriminative peaks when discriminating the different ST types of *S. haemolyticus* based on MALDI-TOF MS spectral data. Therefore, we used a variety of different strategies in order to identify the discriminative peaks that are very likely to be highly related to the different ST types. An exploration of the discriminative peaks is highly dependent on the feature selection strategy and the machine learning method. It is important to note that the performance of RF is relatively robust and that it is also less time-consuming during training; in these circumstances, we largely adopted feature selection using RF for this study. The stepwise strategy is similar to the brute force method when used to find the best combinations for the classifiers. Consequently, the results of the stepwise strategy are generally better than those of the forward strategy. Furthermore, there is only one model that did not include peak 4673, which strongly supports peak 4673 as a discriminative peak. In addition, peak 5129 was not selected by two models, as shown in Figure 8, indicating that the normalized intensities across the three groups for this peak are apparently different. In addition, both Figure 8 and Table 3 show that the occurrence ratio is also significantly different across the three groups. Specifically, ST42 rarely presented peaks 4673 and 5129, while ST3 usually presented peaks at m/z 4673 and 5129. Further experiments are needed to identify the peptides corresponding to these peaks.

Although the machine learning-based classifiers has demonstrated impressive performance in this study for distinguishing different ST types of *S. haemolyticus*, there are still some limitations. One major concern is that subspecies composition of the microbial strains may differ in different bacterial populations or in different regions of the world. In such circumstances the construction of machine learning-based classifier-based method might break down because these groups have different discriminative peaks for these subspecies. Even so, the machine learning-based classifier approach, in conjunction with the associated statistical tests, still provides a novel framework for analyzing MALDI-TOF MS data. Another critical issue that has been identified in the previous studies is the reproducibility of the mass spectra when MALDI-TOF MS is being used in bacterial typing (Walker et al., 2002; Wolters et al., 2011; Croxatto et al., 2012; Sandrin et al., 2013). There are a variety of factors involved in the reproducibility of the mass spectra and these include sample processing and specimen type (Josten et al., 2013; Sandrin et al., 2013; Mather et al., 2016). As of yet no standard protocol has been proposed for strain typing by MALDI-TOF MS. Nevertheless, a standard protocol should be optimized and specified for each species in order to achieve a robust performance when strain typing (Walker et al., 2002; Sandrin et al., 2013). The College of American Pathologists accreditation and proficiency test has been conducted for years to ensure the performance and quality standards of personnel and tests at Chang Gung Memorial Hospital, Linkou Branch. Therefore, on the basis of previous qualified MALDI-TOF MS workflow and data used here, the constructed classification models used in this study are readily available for *S. haemolyticus* strain typing.

Our study has demonstrated a method of developing robust classifiers for discriminating different ST types of *S. haemolyticus* based on MALDI-TOF MS data. The multi-class classifier demonstrated an AUC of 0.848 and accuracy of 0.886 when discriminating these three groups. If we only consider binary classification for ST3 and ST42, the AUC reaches an excellent discrimination power of 0.972. The constructed classifiers were able to provide instant information when identifying the origin of infection, which will allow rapid infection control. As a result, we believe that we have hereby developed a cost effective and rapid identification method for the strain typing of *S. haemolyticus*. This provides a great opportunity for further improvement of this new protocol and its introduction into routine clinical microbiology laboratory practices in order to attain rapid infection control. Furthermore, the explicit strategy for the determination of representative peaks before constructing the classifiers provides some indications for those who are interested in further analysis of spectra data.

## Data Availability

The raw data supporting the conclusions of this manuscript will be made available by the authors, without undue reservation, to any qualified researcher.

## Author Contributions

H-YW carried out the data collection and curation. C-RC participated in the data analyses, model construction, and drafted the manuscript. C-RC, H-YW, FL, Y-JT, C-HC, T-PL, and T-YL participated in the design of the study and performed the draft revision. J-TH, T-YL, and J-JL conceived of the study, and participated in its design and coordination and helped to revise the manuscript. All authors read and approved the final manuscript.

### Conflict of Interest Statement

The authors declare that the research was conducted in the absence of any commercial or financial relationships that could be construed as a potential conflict of interest.
