# Supplementary material for: Incorporating Statistical Test and Machine Intelligence Into Strain Typing of Staphylococcus haemolyticus Based on Matrix-Assisted Laser Desorption Ionization-Time of Flight Mass Spectrometry
Source: Front Microbiol. 2019 Sep 13;10:2120. doi: 10.3389/fmicb.2019.02120 (PMC6753874; doi:10.3389/fmicb.2019.02120)
Supplement: Supplementary file 1 [file Data_Sheet_1.PDF]

Supplementary Material

0001	
**m/z**	**Intensity**
2277.222	0.195
2383.227	0.229
**2530.531**	0.313
**2533.844**	0.272
2636.336	0.297
2652.109	0.568
⋮	⋮

0002	
**m/z**	**Intensity**
2386.525	0.128
**2553.633**	0.105
**2558.731**	0.152
2663.817	0.206
2836.139	0.458
3152.862	0.357
⋮	⋮

…

0154	
**m/z**	**Intensity**
**2636.921**	0.095
**2639.865**	0.181
2858.138	0.403
3013.215	0.438
3326.592	0.787
3580.193	0.181
⋮	⋮

NO	…	2380	2532	2550	2638	2660	…	Label
0001	…	0.229	0.313	0	0.297	0.568	…	Type 3
0002	…	0.128	0	0.105	0.206	0.206	…	Type 3
⋮	⋮	⋮	⋮	⋮	⋮	⋮	⋮	
0154	…	0	0	0	0.181	0	0	Others

Supplementary Figure 1. Illustration of the spectrum data preprocessing. The data in the red border represents the original spectral data, and the following form can be used directly for constructing the classifiers.

Supplementary Table 1. Number of other ST types of *S. haemolyticus*.

ST types	Number of isolates	Average of number of detected peaks
ST1	9	66.89
ST8	1	64.00
ST9	5	79.40
ST23	3	77.00
ST25	4	79.75
ST29	9	74.56
ST30	5	81.00
ST43	2	87.00
ST44	3	67.00
ST45	1	68.00
ST46	1	65.00
ST47	2	53.50
ST48	2	91.00
